# Endocytosis, trafficking and exocytosis of intact full-length botulinum neurotoxin type a in cultured rat neurons

**DOI:** 10.1016/j.neuro.2020.02.009

**Published:** 2020-05

**Authors:** Luis Solabre Valois, Kevin A. Wilkinson, Yasuko Nakamura, Jeremy M. Henley

**Affiliations:** School of Biochemistry, Centre for Synaptic Plasticity, Biomedical Sciences Building, University of Bristol, Bristol, BS8 1TD, UK

**Keywords:** Neuron, Botulinum neurotoxin type A, BoNT/A, Degradation, Endocytosis

## Abstract

Botulinum toxin A (BoNT/A) is a potent neurotoxin that acts primarily by silencing synaptic transmission by blocking neurotransmitter release. BoNT/A comprises a light chain (LC/A) intracellular protease and a heavy chain (HC/A) composed of a receptor binding domain (HC_C_/A) and a translocation domain (HC_N_/A) that mediates cell entry. Following entry into the neuron, the disulphide bond linking the two peptide chains is reduced to release the LC/A. To gain better insight into the trafficking and fate of BoNT/A before dissociation we have used a catalytically inactive, non-toxic full-length BoNT/A(0) mutant. Our data confirm that BoNT/A(0) enters cortical neurons both in an activity-dependent manner and via a pathway dependent on fibroblast growth factor receptor 3 (Fgfr3) signalling. We demonstrate that both dynamin-dependent endocytosis and lipid rafts are involved in BoNT/A internalisation and that full-length BoNT/A(0) traffics to early endosomes. Furthermore, while a proportion of BoNT/A remains stable in neurons for 3 days, BoNT/A degradation is primarily mediated by the proteasome. Finally, we demonstrate that a fraction of the endocytosed full-length BoNT/A(0) is capable of exiting the cell to intoxicate other neurons. Together, our data shed new light on the entry routes, trafficking and degradation of BoNT/A, and confirm that trafficking properties previously described for the isolated HC_C_/A receptor binding domain of are also applicable to the intact, full-length toxin.

## Highlights

• Full length catalytically inactive BoNT/A(0) enters neurons by at least two routes.
• Internalised BoNT/A(0) traffics to early endosomes.
• A proportion of internalised BoNT/A remains stable in neurons for 3 days.
• Endocytosed full-length BoNT/A(0) can exit the cell to intoxicate other neurons.

## 1 Introduction

The clostridial neurotoxin (CNT) family of proteins includes several serotypes of BoNTs and tetanus neurotoxin (TeNT) (Arnon et al., 2001; Montecucco and Schiavo, 1994). CNTs comprise a ∼50 kDa Light Chain (LC) and a ∼100 kDa Heavy Chain (HC) connected by a disulfide bond (Lacy et al., 1998; Montal, 2010; Montecucco and Schiavo, 1994). The LC is a metalloprotease that cleaves proteins of the Soluble N-ethylmaleimide-sensitive factor Attachment Protein Receptor (SNARE) complex, blocking synaptic vesicle exocytosis (Schiavo et al., 1994). The HC is further divided into two functional domains: the C-terminal receptor-binding domain (HC_C_) mainly responsible for endocytosis and trafficking, and an N-terminal pore-forming domain (HC_N_), which allows the translocation of the LC into the cytosol once it has been endocytosed (Dong et al., 2018).

Intoxication by a CNT can be summarised in three steps: *i)* HC_C_ binds to the cell membrane and is internalised into an endocytic compartment; *ii)* HC_N_ domain forms a channel in the endocytosed vesicle membrane; *iii)* LC translocates through HC_N_ to the cytosol, separates from the HC and targets SNARE proteins, blocking synaptic vesicle release (Dong et al., 2018).

This functional separation of BoNT/A domains has generated valuable information on the processes underlying BoNT/A action. For example, BoNT/A trafficking has been extensively investigated using the isolated receptor-binding domain (HC_C_/A). HC_C_/A binds to receptor proteins and gangliosides at the neuronal surface to facilitate its incorporation into several different pools of synaptic vesicles (Harper et al., 2016; Pellett et al., 2015; Restani et al., 2012b). HC_C_/A then enters the endocytic pathway where most progresses on to autophagosomes but a fraction remains stable in early endosomes (Colasante et al., 2013; Couesnon et al., 2009; Harper et al., 2011). From early endosomes it can be retrogradely trafficked, exocytosed and taken up by surrounding cells (Bomba-Warczak et al., 2016; Restani et al., 2012b; Wang et al., 2016). In addition, a proportion of HC_C_/A molecules also traffic to lysosomes for degradation (Harper et al., 2011; Wang et al., 2015a, 2015b).

Notwithstanding this progress using the specific HC_C_/A domain as a tool, it remains unclear if it faithfully reports the fate of full-length BoNT/A. This issue is highlighted by the observation that the corresponding domain of TeNT, HC_C_/T, is trafficked differently to full-length TeNT and isolated fragments of the toxin (Blum et al., 2012; Ovsepian et al., 2015), and other groups have reported that both HC_N_/A and LC/A are involved in trafficking (Ayyar et al., 2015; Montecucco et al., 1988).

To investigate the trafficking and fate of full-length BoNT/A prior to dissociation we have used BoNT/A(0), a catalytically inactive and non-toxic full-length point mutant, to follow the endocytosis, trafficking and degradation of the toxin. Our rationale is that the trafficking of this point mutated full-length toxin protein should be more informative than looking at individual toxin subunits or fragments, which have been used previously. Furthermore, the fact that is safe to use and relatively easy to produce makes it an amenable tool for labs that do not have high-level biosecurity and containment facilities or the licencing to produce and investigate the fully active toxin.

Our data show that BoNT/A(0) enters neurons via activity-dependent and Fgfr3-mediated routes that involve both dynamin and lipid rafts. Once internalised, BoNT/A(0) traffics through early endosomes but escapes lysosomal degradation, being largely degraded by the proteasome. However, a fraction of internalised BoNT/A(0) is stable for at least 3 days in neurons. Finally, we demonstrate that a fraction of intact BoNT/A(0) can be exocytosed from neurons and enter surrounding cells.

## 2 Materials and methods

### 2.1 Primary neuronal cultures

Dissociated hippocampal and cortical neuronal cultures were prepared as previously described (Carmichael et al., 2018; Martin and Henley, 2004). Briefly, pregnant Wistar rats were sacrificed by schedule 1 lethal anaesthesia, following procedures in full compliance with ARRIVE guidelines and the U.K. Animals Scientific Procedures Act, 1986. Neurons were dissected from E18 Wistar rat pups followed by trypsin dissociation and cultured for up to 2 weeks. For the first 24 h, cells were grown in plating media: Neurobasal media (Gibco) supplemented with 5% horse serum (Sigma), B27 (1x, Gibco), P/S (100 units penicillin and 0.1 mg/ml streptomycin; ThermoScientific) and 5 mM Glutamax (Gibco). After 24 h, plating media was replaced with feeding media (same composition as plating medium but containing 2 mM Glutamax and lacking horse serum). For biochemistry experiments, cells were plated at a density of 500,000 per 35 mm well and 250,000 per coverslip for imaging experiments. Animal care and procedures were carried out in accordance with UK Home Office and University of Bristol guidelines.

### 2.2 Production of recombinant BoNT/A(0)

BoNT/A(0), containing two point mutations, E224Q/H227Y, that render it catalytically inactive (Kukreja et al., 2007; Zhou et al., 1995), was supplied by Ipsen as a single chain polypeptide using their standard protocols. The amino acid sequence of BoNT/A(0) is shown in Supplementary Fig. 1.

**Fig. 1:**
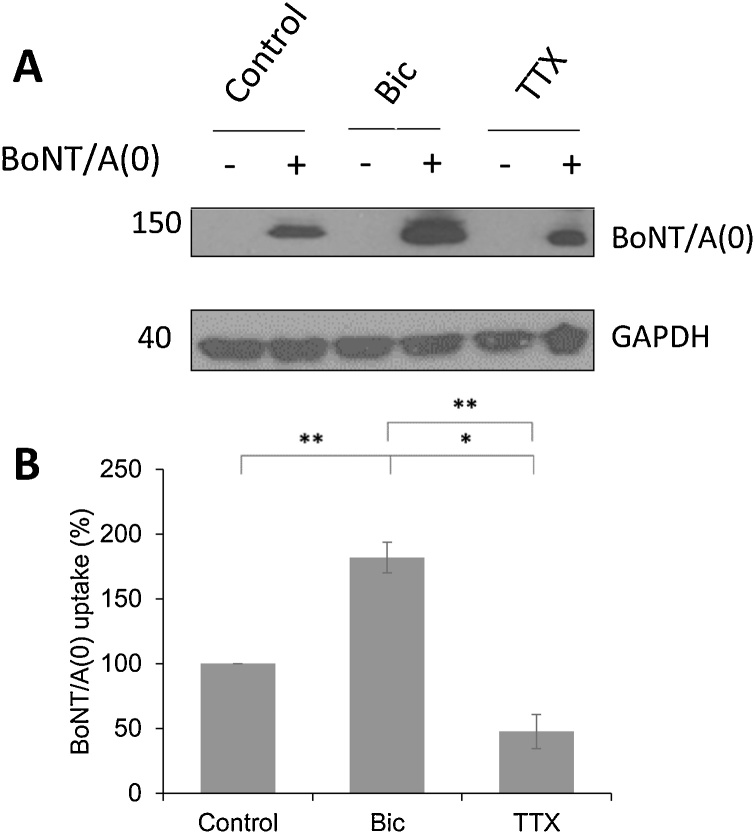
Modulation of neuronal activity regulates BoNT/A(0) uptake into cortical neurons. A) Representative western blots of full-length BoNT/A in DIV14-17 cortical neurons pre-treated for 1 h with vehicle (0.1 % DMSO), 1 h with 1 μM bicuculline or 18 h with 2 μM TTX followed by 25 nM BoNT/A(0) for 10 min. GAPDH was used as a loading control. B) Quantification of the western blot results. Mean values ± SEM analysed using ANOVA followed by Tukey *post hoc* test, *p < 0.05, **p < 0.01. N = 3.

### 2.3 Treatments

Cultures were exposed to 25 nM of heterodimeric BoNT/A(0) for 10 min unless indicated otherwise and all incubations were done at 37 °C. The IC_50_ for BoNT/A cleavage of SNAP-25 is in the pM range but BoNT/A(0) reaches a binding/internalisation plateau at ∼25 nM (Vazquez-Cintron et al., 2014). Thus, to enable immuno-detection of the toxin it is standard in the field to use nM concentrations (Harper et al., 2011, 2016; Restani et al., 2012b; Wang et al., 2015a, 2015b).

Where indicated, TTX (2 μM) was applied 18 h before BoNT/A(0) to suppress synaptic transmission. Bicucculine (1 μM) was applied 1 h before BoNT/A(0) to block inhibitory GABA_A_ receptors and enhance synaptic transmission.

The Fgfr3 inhibitor SU5402 (20 μM), the dynamin inhibitor MiTMAB (25 μM), the cholesterol-extracting agent methyl-β-cyclodextrin (MCD; 1 mM), the lysosomal degradation blocker leupeptin (30 μM), and proteasomal degradation blocker MG132 (5 μM) were diluted to working concentrations in feeding medium.

For pulse-chase experiments, neurons were treated for 10 min with BoNT/A(0), washed and immediately fixed (designated as 0 min) or incubated for a further 10 min or 60 min prior to fixing. A further control not exposed to BoNT/A(0) was also included in these experiments. Neurons were then immunolabelled with antibodies against BoNT/A and the early endosome marker EEA1.

For stability studies, cells were incubated in pre-warmed feeding medium with BoNTA(0) for 10 min and were then washed three times with PBS. Their media was replaced, and cells were incubated for the times indicated.

For degradation studies, cells were incubated in medium containing the desired proteolysis inhibitors for 30 min before applying BoNT/A(0). After the 30 min the inhibitor media was removed and pre-warmed feeding media containing both the toxin and the relevant proteolysis inhibitor at the concentration specified was applied to the corresponding cultures. Cultures were exposed to BoNT/A(0) for 10 min. Then, the BoNT/A(0)-containing medium was removed, cells washed and media with the respective proteolysis inhibitors was reapplied and left for 18 h in the incubator.

### 2.4 SDS-PAGE and western blotting

Treated cells were washed thoroughly with 0.1 M glycine, 0.1 M HCl, and then with PBS before lysing in SDS-PAGE loading buffer. Proteins were separated by SDS-PAGE under non-reducing conditions to preserve the integrity of the disulfide bond connecting LC and HC and transferred to PVDF membrane for western blotting. Membranes were blocked in 5% (w/v) non-fat milk powder in PBS-T. Antibodies were diluted 1:10000 (GAPDH from Abcam, Cat. No. ab8245) and 1:600 (BoNT/A from Ipsen). Western blots were imaged using X-ray films in a dark room using developer and fixer solutions. The blots were then scanned and quantified by densitometry using FIJI (ImageJ studio). Because we needed to use the minimal dose possible for *in vitro* experiments, combined with the relatively short incubation times, we worked at the limits of detection. Thus, although in some examples a higher molecular weight band is observed, in all cases the ∼150 kDa BoNT/A(0) band was used for densitometric quantification, which was consistently reproducible between experiments. In all cases, intensities for BoNT/A(0) signal were normalised to the GAPDH signal from the same sample.

### 2.5 Immunofluorescence

Cells were washed with phosphate buffered saline (PBS) and fixed with pre-warmed 4% paraformaldehyde (PFA) for 10 min at room temperature. Coverslips were incubated with PBS with 3% (w/v) bovine serum albumin (BSA) and 0.1 % Triton X-100 for 20 min at room temperature to block and permeabilise neurons. Antibodies were mixed with PBS containing 3% (w/v) BSA to their appropriate working concentrations. Custom-made polyclonal anti-BoNT/A antibodies targeting the full-length toxin (Eurogentec) were diluted at 1:500, EEA1 antibodies (BD Biosciences, Cat. No. 610457) were diluted 1:200. These were incubated at 4 °C overnight. Coverslips were then washed and incubated with secondary antibodies (Jackson ImmunoResearch) in PBS with 3% (w/v) BSA for 1 h at room temperature. Coverslips were washed and mounted onto microscope slides using Fluoromount-G (Thermo Fischer Scientific, Cat. No 00-4959-52). A Leica SP5-AOBS confocal laser scanning microscope was used for confocal imaging.

### 2.6 Statistical analysis

All data are presented as mean ± SEM. Statistical significance was determined by One-way ANOVA followed by Tukey *post hoc* test or Student’s t-test, as indicated in the figure legends, using Graphpad Prism software. P < 0.05 was considered statistically significant; *p < 0.05, **p < 0.01. The stated N number refers to the number of independent neuronal cultures used for each experiment.

## 3 Results

### 3.1 Synaptic activity modulates BoNT/A(0) entry into neurons

We first confirmed the uptake of BoNT/A(0) by our cultures, and our ability to detect it, by applying BoNT/A(0) to cortical neurons for 10 min followed by western blotting with an anti-BoNT/A antibody. Western blots confirmed that 150 kDa full-length toxin immunoreactive bands were present only in treated neurons (Fig. 1A, control lanes).

We then determined whether full-length BoNT/A(0) internalisation was activity-dependent by treating neuronal cultures with the Na^+^-channel blocker tetrodotoxin (TTX), which prevents action potentials and suppresses synaptic activity (Bane et al., 2014), or with the GABA_A_ receptor (GABA_A_R) antagonist bicuculline (Bic) which, by inhibiting inhibitory synapses, increases the overall activity of the network (Olsen, 2018). Neurons were pre-treated with either vehicle (dimethyl sulphoxide, DMSO), TTX or Bic as specified before treatment with 25 nM BoNT/A(0) for 10 min prior to lysis. Toxin uptake was assessed by western blotting.

Densitometry of the western blots showed that BoNT/A(0) uptake was 181.8 ± 11.7 % in Bic-treated neurons when compared to the DMSO control (p** < 0.01), while uptake in TTX-treated neurons was reduced to 47.5 ± 13.2 % of control (p** < 0.01; Fig. 1**A,B**). These data confirm that a substantial proportion of BoNT/A(0) is internalised in a neuronal activity-dependent manner. However, we also observed that TTX treatment did not completely block toxin uptake, raising the possibility of other potential routes of entry.

### 3.2 A proportion of BoNT/A(0) internalisation requires Fgfr3 signalling

**Fig. 2:**
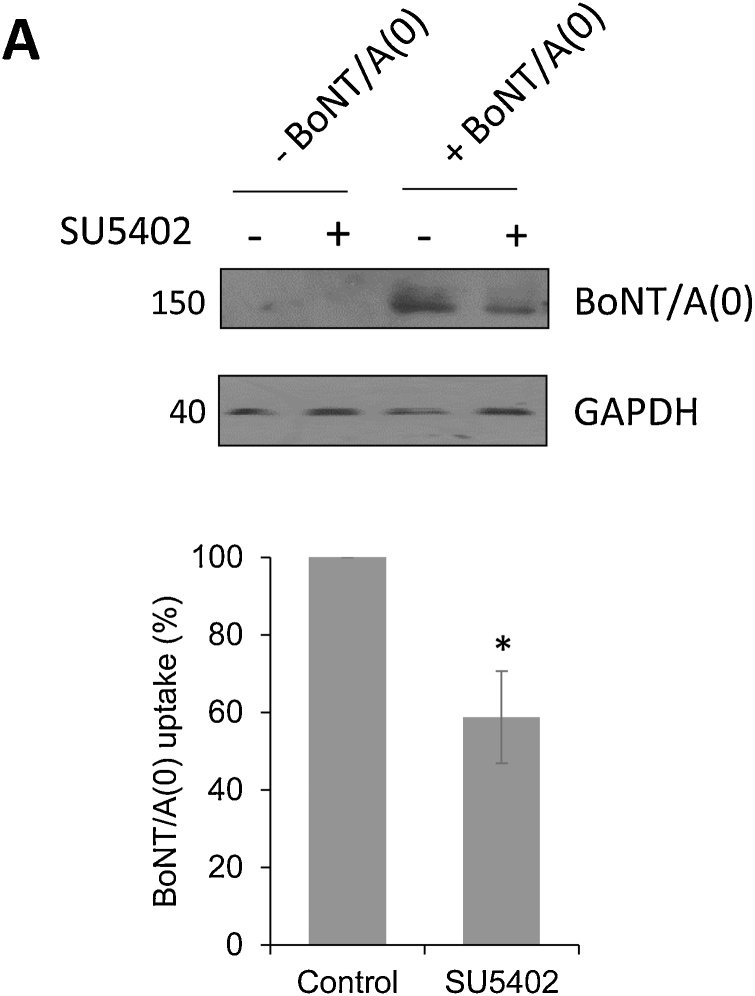
Blocking Fgfr3 activity significantly decreases BoNT/A(0) uptake into cortical neurons. A) Representative western blot of samples from DIV14-17 cortical neurons pre-treated for 1 h with vehicle (0.1 % DMSO) or 20 μM SU5402 before incubation for 10 min with 25 nM BoNT/A(0) in the presence of this compound, plus non-treated controls. Membranes were probed with anti-BoNT/A and anti-GAPDH antibodies. B) Quantification of the results, represented as mean values ± SEM. Student’s t-test, *p < 0.05. N = 3.

Fibroblast growth factor receptor 3 (Fgfr3) is a tyrosine kinase receptor that has been reported to bind to, and be activated by, HC_C_/A in neuroblastoma cells (Jacky et al., 2013). To determine whether Fgfr3 contributes to BoNT/A(0) uptake in primary neurons, we used SU5402, which inhibits Fgfr3 phosphorylation, down-stream signalling and internalisation (Lamont et al., 2011). In cortical cultures pre-treated with SU5402 for 1 h and then incubated with 25 nM BoNT/A(0) for 10 min, BoNT/A(0) uptake was significantly reduced compared to control (58.8 ± 11.9 %; p* < 0.05; Fig. 2). These data suggest that, in addition to synaptic vesicle-mediated entry, a proportion of full-length BoNT/A depends on Fgfr3 signalling to enter neurons.

### 3.3 BoNT/A(0) uses dynamin-dependent endocytosis and lipid rafts to enter neurons

We next investigated the mechanisms of BoNT/A(0) internalisation. To do this, we used myristyl-trimethyl-ammonium bromide (MiTMAB), a dynamin I and dynamin II inhibitor that competitively interferes with the ability of dynamin to bind phospholipids and prevents receptor-mediated endocytosis (Quan et al., 2007). We also examined the effects of methyl-β-cyclodextrin (MβCD), a complexing agent that depletes cholesterol from membranes, specifically disrupting lipid rafts while leaving other endocytic mechanisms unaffected at the concentrations used (Mahammad and Parmryd, 2015; Rodal et al., 1999).

**Fig. 3:**
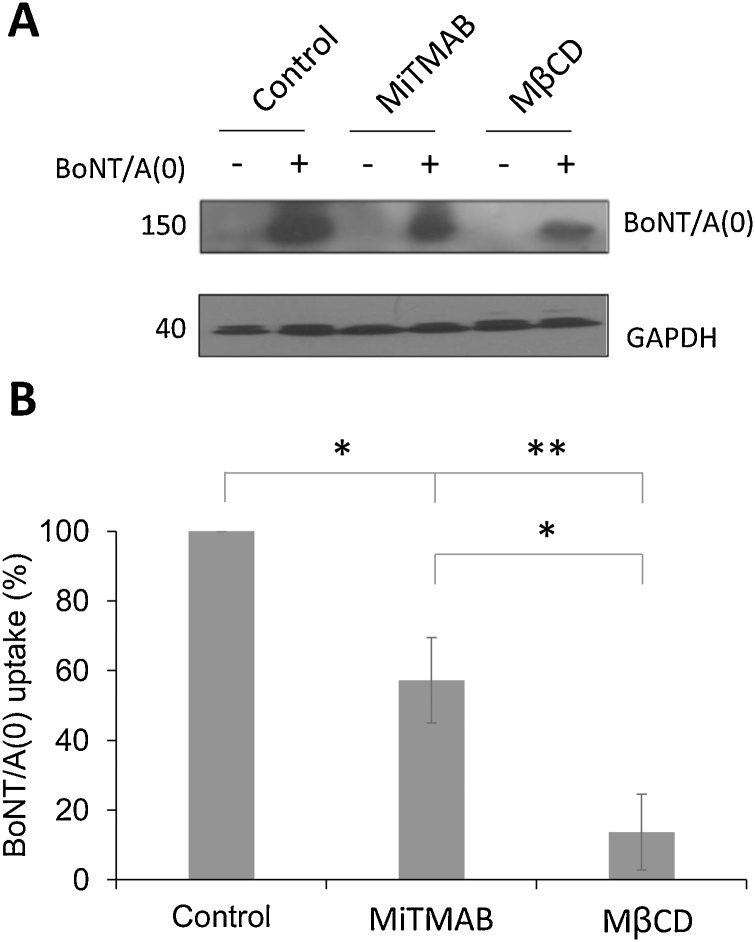
Methyl-β-cyclodextrin and MiTMAB inhibit BoNT/A(0) uptake. A) Representative western blot of samples from DIV14-17 cortical neurons pre-treated for 30 min with vehicle (0.1 % DMSO), 25 μM MiTMAB or 1 mM methyl-β-cyclodextrin before incubation for 10 min with 25 nM BoNT/A(0) in the presence of these compounds, plus non-treated controls. Membranes were probed with anti-BoNT/A and anti-GAPDH antibodies. B) Quantification of the results, represented as mean values ± SEM. ANOVA followed by Tukey *post hoc* test, *p < 0.05, **p < 0.01. N = 3.

Cells were pre-treated with endocytosis inhibitors for 30 min and exposed to BoNT/A(0) for 10 min. Both inhibitors caused a significant decrease in BoNT/A(0) endocytosis (Fig. 3). In MiTMAB-treated neurons, internalisation of BoNT/A(0) was reduced to 57.2 ± 12.2 % of the control (p* < 0.05), whereas MβCD reduced BoNT/A endocytosis to 13.6 ± 10.9 % (p**< 0.01). These data suggest that BoNT/A(0) enters neurons via both dynamin-dependent and dynamin-independent endocytosis.

### 3.4 BoNT/A(0) traffic through early endosomes in neurons is limited

To investigate the progression of BoNT/A(0) traffic into the endosomal pathway we used a pulse-chase protocol followed by staining for internalised BoNT/A(0) and the early endosomal marker EEA1.

**Fig. 4:**
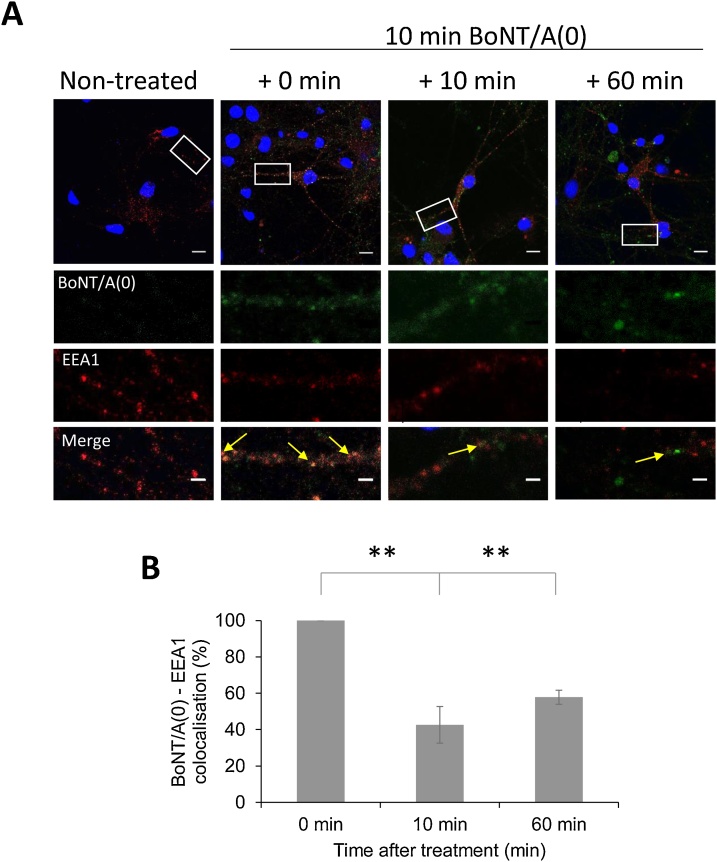
BoNT/A(0) traffics through early endosomes. A) Representative images of DIV14-17 hippocampal neurons treated with 25 nM BoNT/A(0) for 10 min. After toxin exposure, cells were washed and internalised toxin allowed to traffic for 0 min, 10 min or 1 h. Cells were treated at different time points and fixed at the same time. Staining corresponds to EEA1 (red) and BoNT/A(0) (green) and DAPI (blue). Scale bars are 1 μm. The lower 3 rows of panels show enlargements of the boxed area in the top panels for BoNT/A(0) (green), EEA1 (red) and colocalization (yellow), respectively. B) Quantification of the results, represented as mean values ± SEM. Colocalisation between BoNT/A(0) and EEA1 measured using Manders’ coefficient and normalised to the value at time 0. ANOVA followed by Tukey *post hoc* test, **p < 0.01. N = 3.

In neurons fixed immediately after being treated with BoNT/A(0) for 10 min (t = 0) the Manders’ coefficient of BoNT/A(0) co-localisation with EEA1 was M = 0.591 ± 0.112, indicating positive but incomplete trafficking through early endosomes (Fig. 4). Values for the later time points were normalised to this value. In cells fixed 10 min after the end of the 10 min BoNT/A(0) incubation period co-localisation was reduced to 42.6 ± 6.8 % of t = 0 (**p < 0.01). This fraction remained relatively constant, with a value of 57.8 ± 3.8 % after 60 min (**p < 0.01). These data indicate that BoNT/A(0) very rapidly traffics into early endosomes and ∼50 % exits within 10 min. However, colocalisation levels remain stable 1 h after treatment.

### 3.5 Heterodimeric BoNT/A(0) is degraded to a stable level

**Fig. 5:**
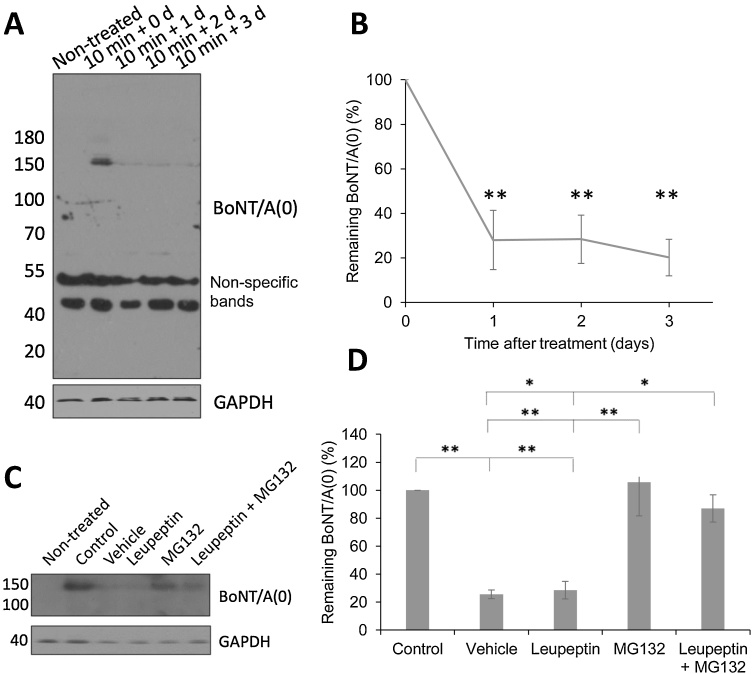
BoNT/A(0) is degraded to a stable level by the proteasome. A) Representative western blot of lysates from DIV14-17 cortical neurons treated with 25 nM BoNT/A(0) for 10 min, after which they were washed and their corresponding conditioned medium replaced and left for different times. Cells were treated on different days and lysed at the same time. Membranes were probed with anti-BoNT/A and anti-GAPDH antibodies. B) Quantification of the data in A. Mean ± SEM. ANOVA followed by Tukey *post hoc* test, ** = p < 0.01. N = 4. C) Representative western blot of samples from DIV14-17 cortical neurons pre-treated for 30 min with vehicle (0.1 % DMSO), 30 μM leupeptin, 5 μM MG132, or a combination of both, before a 10 min treatment with 25 nM BoNT/A(0) in the continued presence of these compounds. Cells were then washed and replaced in the corresponding medium containing proteolysis inhibitors for 18 h. Control cells were exposed to 25 nM BoNT/A(0) for 10 min and then lysed immediately. Other conditions were normalised to these values. Vehicle corresponds to DMSO-treated cells without degradation inhibitors. Membranes were probed with anti-BoNT/A and anti-GAPDH antibodies. D) Quantification of the results in C. Mean values ± SEM. ANOVA followed by Tukey *post hoc* test, * = p < 0.05, ** = p < 0.01. N = 3.

It has been reported that LC/A is stable in neuronal cytoplasm for prolonged periods (Tsai et al., 2017), but to determine the time course of full-length BoNT/A(0) degradation, we treated neurons for 10 min with BoNT/A(0) and then washed and returned to conditioned media for 0 (lysed immediately), 1, 2 or 3 days. Western blot analyses with the polyclonal antibody, which recognises both full-length ∼ 150 kDa BoNT/A and the ∼100 kDa HC/A fragment indicated that after 1 day, 28.0 ± 13.3 % of the originally internalised BoNT/A(0) was present inside the cells (**p < 0.01), and this amount remained constant 3 days after treatment (Fig. 5A,B; 28.3 ± 10.9 % after 2 days, 20.1 ± 8.3 % after 3 days, **p < 0.01 in both cases). Since no ∼100 kDa HC/A band was detected in these cells, these data suggest that if the full-length BoNT/A is cleaved the HC/A fragment is degraded. Thus, although most full-length BoNT/A(0) is degraded (either before or after cleavage into the HC/A and LC/A fragments) within 24 h, ∼30 % of the endocytosed full-length BoNT/A(0) remains intact and is stable for at least 3 days.

### 3.6 BoNT/A(0) is degraded by the proteasome and not the lysosome

To investigate how heterodimeric BoNT/A(0) is degraded in neurons, we pre-treated cortical neurons with the lysosomal inhibitor leupeptin, the proteasomal inhibitor MG132, or both, for 30 min before applying 25 nM BoNT/A(0) for 10 min. Neurons were then washed and incubated for a further 18 h in the continued presence of the inhibitors. A positive control with cells treated for 10 min with 25 nM BoNT/A(0) and DMSO was included to assess the levels of protein before degradation.

In the absence of leupeptin or MG132 25.5 ± 3.0 % of endocytosed ∼150 kDa BoNT/A(0) remained intact after 18 h incubation compared to the control (p** < 0.01; Fig. 5C,D). Similarly, in leupeptin-treated neurons 28.6 ± 6.3 % of the original BoNT/A(0) remained after 18 h (p < 0.01), suggesting full-length BoNT/A(0) is not subject to lysosomal degradation. In contrast, MG132, alone, or in combination with leupeptin, effectively prevented full-length BoNT/A(0) degradation, suggesting that BoNT/A(0), like Fgfr3 (Jacky et al., 2013), is primarily degraded via the proteasome.

### 3.7 Intact BoNT/A(0) can be exocytosed to enter surrounding neurons

**Fig. 6:**
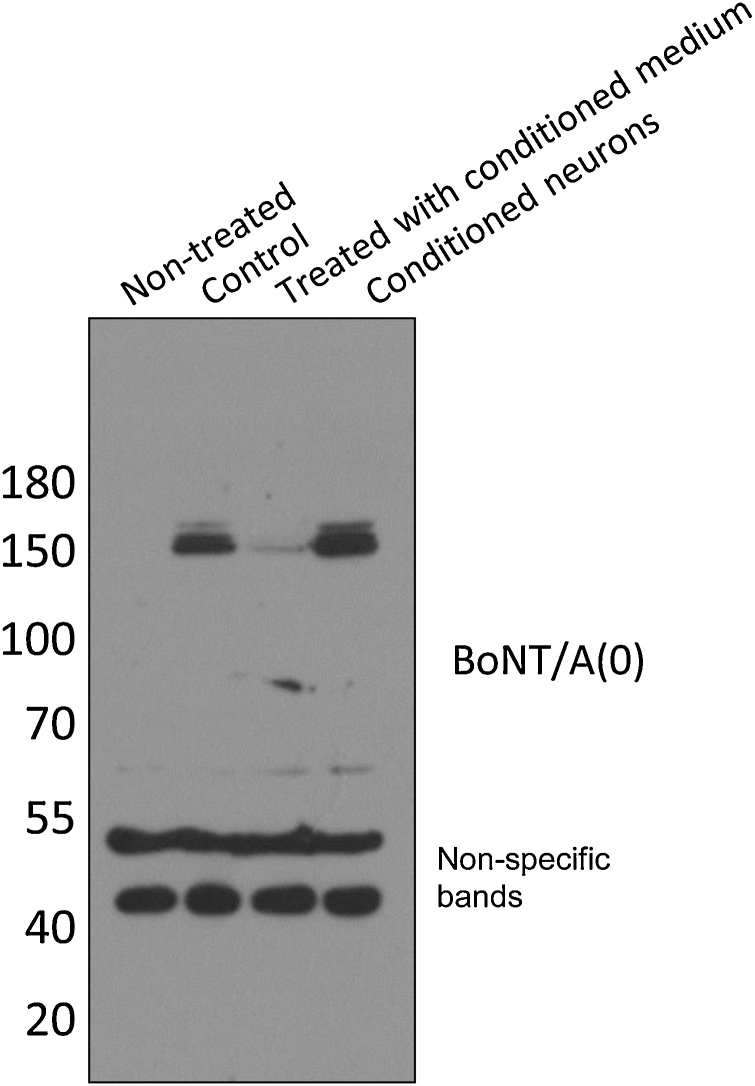
BoNT/A(0) is internalised from medium conditioned by BoNT/A(0)-treated neurons. DIV14-17 cortical neurons were treated with 25 nM BoNT/A(0) for 18 h (fourth lane). After this time, cells were washed and medium was replaced with fresh feeding medium and incubated for 24 h. Following this, conditioned medium from cultures in the fourth lane was removed and applied to non-treated cells in third column and incubated for 24 h. Cells in the first and second lanes received fresh feeding medium. Neurons treated for 10 min with 25 nM BoNT/A(0) at the end of the experiment were included as a positive control (second lane), and non-treated cells as a negative control (first lane). Cells were lysed on the same day and their lysates analysed by western blot. Membranes were probed with anti-BoNT/A and anti-GAPDH antibodies. N = 3.

We next investigated the fate of non-dissociated, non-degraded BoNT/A(0). DIV14-17 cortical neurons were treated with 25 nM BoNT/A(0) for 18 h. Following this incubation period, the neurons were washed and medium was replaced with fresh feeding medium and the cells incubated for further 24 h. The conditioned medium from the washed previously intoxicated cells was then removed and applied to fresh neurons that had not been previously exposed to BoNT/A(0). These neurons were incubated in the conditioned media for 24 h. As a negative control, neurons from the same dissection were untreated and kept in the same incubator for 66 h. A positive intoxication control was that another batch of previously untreated neurons from the same dissection were exposed 25 nM BoNT/A(0) for 10 min at the end of the experiment. No gross changes in neuronal morphology were observed under any condition. Neurons from all conditions were extensively washed and lysed for western blotting at the same time. As shown in Fig. 6, neurons exposed to conditioned media for 24 h contain BoNT/A(0) similar to the positive control neurons which were exposed to 25 nM BoNT/A(0) for 10 min. These results are consistent with intact BoNT/A(0) being released from neurons after endocytosis and entering fresh, previously non-intoxicated neurons.

## 4 Discussion

### 4.1 BoNT/A(0) enters neurons via activity- and Fgfr3-dependent mechanisms

We show that enhancing neuronal network activity increases, whereas blocking neuronal activity reduces, BoNT/A(0) endocytosis into neurons. These results confirm that BoNT/A(0) enters cortical neurons via activity-dependent pathways consistent with a primary route for internalisation via synaptic vesicles (Colasante et al., 2013; Kristensson and Olsson, 1978). Interestingly, however, the initial binding of BoNT/A to gangliosides in the cell membrane is independent of neuronal activity (Simpson, 1980). Furthermore, in our experiments, in which neurons were thoroughly washed to exclude the possibility of a signal from surface bound but not internalised BoNT/A(0), TTX blockade did not completely prevent BoNT/A(0) uptake, indicating additional routes of entry. These findings raise the possibility of alternative routes for BoNT/A(0) entry independent of synaptic vesicles, or that in the presence of TTX there is a basal level of constitutive synaptic vesicle endocytosis. Although challenging, further work using specific inhibitors of synaptic vesicle uptake, to determine the exact contribution of the synaptic vesicle cycle to full-length toxin internalisation, would undoubtedly benefit the field.

In clonal cells, HC_C_/A has been reported to act as a ligand for Fgfr3 (Jacky et al., 2013), which undergoes dynamin-independent endocytosis (Haugsten et al., 2011). Here we show that the Fgfr3 blocker SU5402 reduces BoNT/A(0) endocytosis in neurons, which is supportive of a role for Fgfr3 in BoNT/A(0) internalisation in neurons.

Inhibition of dynamin-dependent endocytosis by MiTMAB also impaired BoNT/A(0) endocytosis. However, we cannot exclude that, rather than being due to direct inhibition of dynamin-dependent vesicle endocytosis (Bartolome-Martin et al., 2012), these effects could be explained by alternative routes exploited by BoNT/A(0) that were indirectly affected by MiTMAB. Depletion of membrane cholesterol with the cyclodextrin MβCD also dramatically reduced BoNT/A(0) uptake in our neurons, suggesting a role for lipid rafts in toxin internalisation. Indeed, it has been reported that HC_C_/A binds to lipid rafts in neurons (Herreros et al., 2001) and that in motor neuron-like NG108-15 cells, MβCD reduces HC_C_/A binding to the cell membrane (Couesnon et al., 2009). Furthermore, in differentiated PC-12 cells and spinal cord motor neurons, the related TeNT protein uses lipid rafts to enter cells (Deinhardt et al., 2006; Herreros et al., 2001; Munro et al., 2001), although BoNT/A trafficking differs from that of TeNT (Cai et al., 2017; Lawrence et al., 2012; Wang, F. et al., 2015). In contrast, however, other studies have suggested that MβCD can potentiate BoNT/A intoxication. For example, neonatal mice, which are resistant to BoNT/A, can be sensitised to intoxication using MβCD (Thyagarajan et al., 2017). Furthermore, BoNT/A activity can be enhanced by MβCD in undifferentiated Neuro2A cells (Petro et al., 2006). While the exact reasons for these differences in effect of MβCD remain unclear, it is possible that they may reflect differences in the lipid composition of different neuronal preparations.

### 4.2 BoNT/A(0) enters the endocytic pathway in neurons

BoNT/A can enter neurons via synaptic vesicles, from where it traffics to endosomes and autophagosomes (Couesnon et al., 2009; Harper et al., 2011, 2016; Restani et al., 2012a). We detected significant co-localisation of endocytosed BoNT/A(0) with early endosomes and a fraction of BoNT/A(0) remained stable after intoxication, as has been reported previously for HC_C_/A (Restani et al., 2012a). In contrast, HC_C_/A entry under depolarising conditions induced by high potassium suggests that the initial level of HC_C_/A colocalisation with early endosomes is larger and stable over a longer period of time (Couesnon et al., 2009; Harper et al., 2011). It should be noted, however, that potassium depolarisation alters the concentration of organelles and trafficking of BoNT/A through the endocytic pathway (Wang, T. et al., 2015).

### 4.3 BoNT/A(0) is degraded to a stable level by the proteasome

HC_C_/A has been suggested to traffic to, and be degraded by, lysosomes (Harper et al., 2011; Wang, T. et al., 2015). Indeed, we show that full-length BoNT/A(0) levels decreased during the first 24 h after treatment, but thereafter remained relatively stable with no significant further degradation or dissociation occurring over 3 days. Interestingly, intact BoNT/A has been detected in blood up to 25 days after intoxication, suggesting it is remarkably stable (Fagan et al., 2009; Sheth et al., 2008). Thus, in addition to the well-established stability of the catalytically active LC, our data suggest there are mechanisms to protect full-length BoNT/A.

One possible mechanism for the stability and prolonged co-localisation of BoNT/A(0) with early endosomes is that intoxication may impair endosome maturation. Such behaviour would require a blockade of the fusion between the phagosome and the lysosome. Indeed, once endocytosed, some bacteria such as *Brucella suis or Salmonella* arrest endosomal maturation to avoid degradation (Kohler and Roy, 2017; Rajaram et al., 2017; Sun et al., 2013). Moreover, there are intriguing similarities between these bacteria and BoNT/A – for example, *Brucella suis* requires gangliosides to enter cells and is dependent on cholesterol (Naroeni and Porte, 2002). Alternatively, it is possible that the prolonged stability of BoNT/A(0) is due to retention at other locations in the endolysosomal system. However, further work will be required to investigate this possibility directly.

It has been reported that in unstimulated neurons a minority of HC_C_/A molecules retrogradely traffic in acidic organelles (Restani et al., 2012a;Wang et al., 2015a, 2015b) and potassium-induced depolarisation promotes HC_C_/A trafficking to the lysosome, likely targeting it for degradation (Wang et al., 2015a, 2015b). In contrast, we show that, under non-stimulated conditions, intact BoNT/A(0) is predominantly degraded by the proteasome. This is consistent with the observation that, once released into the cytosol, the catalytic domain LC/A can be targeted by the ubiquitin-proteasome system, indicating that LC/A, and therefore BoNT/A(0), may contain proteasome-targeting ubiquitination sites (Kuo et al., 2011; Tsai et al., 2017).

### 4.4 BoNT/A(0) is exocytosed and endocytosed as a full-length toxin in neurons

Our data suggest that a proportion of BoNT/A(0) can be exocytosed from treated neurons to enter surrounding neurons. These data raise the possibility of transcytosis in which BoNT/A(0) enters cells and is subsequently released intact into the medium. It can then be re-endocytosed as a full-length toxin. This is consistent with observations that HC_C_/A is retrogradely transported in autophagosomes in neurons (Restani et al., 2012a; Wang et al., 2015a, 2015b) and reports that BoNT/A effects are found away from the injection site (Antonucci et al., 2008; Restani et al., 2012b).

In summary, we propose *i)* BoNT/A exploits various mechanisms to enter neurons, *ii)* that, under basal conditions, intact BoNT/A is primarily degraded by the proteasome, *iii)* that a significant proportion of BoNT/A remains intact in early endosomes and *iv)* that some BoNT/A(0) is exocytosed and re-endocytosed as a full-length toxin. Taken together, our data characterise the entry and trafficking routes of BoNT/A and provide important information regarding the behaviour of the full-length toxin.

## Author contributions

LSV performed all of the experiments. KAW and YN provided reagents, tools, and technical expertise. JMH supervised the project. LSV and JMH wrote the manuscript and all authors contributed to the editing.

## Declaration of Competing Interest

Luis Solabre Valois was funded by a collaborative PhD scholarship from Ipsen and the University of Bristol. Ipsen provided BoNT/A(0) molecules, anti-BoNT/A antibodies and valuable intellectual interaction and advice.
