# Supplementary material for: Endocytosis, trafficking and exocytosis of intact full-length botulinum neurotoxin type a in cultured rat neurons
Source: Neurotoxicology. 2020 May;78:80–7. doi: 10.1016/j.neuro.2020.02.009 (PMC7225749; doi:10.1016/j.neuro.2020.02.009)
Supplement: Supplementary file 1 [file mmc1.pptx]

## Slide 1
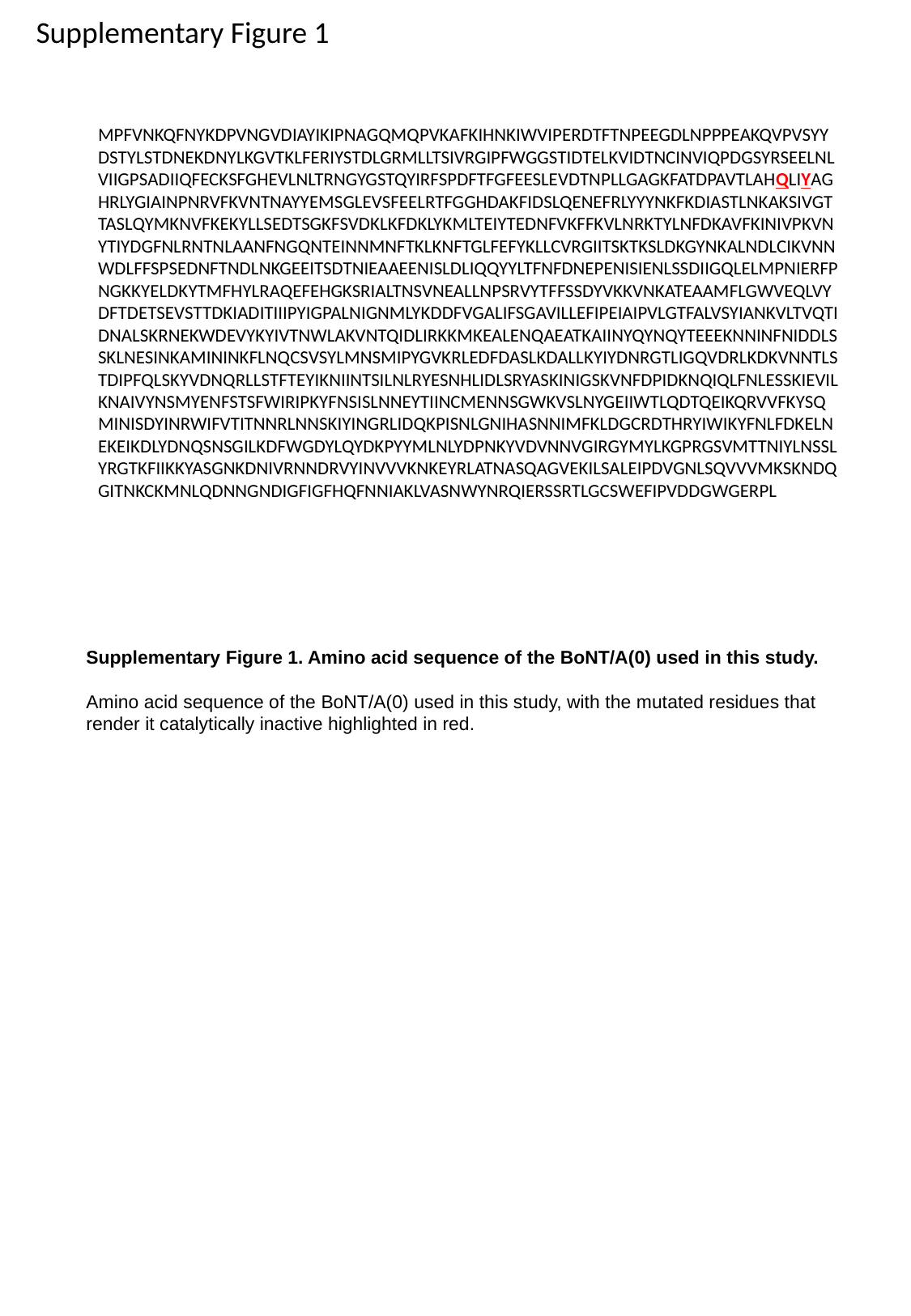

Supplementary Figure 1
MPFVNKQFNYKDPVNGVDIAYIKIPNAGQMQPVKAFKIHNKIWVIPERDTFTNPEEGDLNPPPEAKQVPVSYYDSTYLSTDNEKDNYLKGVTKLFERIYSTDLGRMLLTSIVRGIPFWGGSTIDTELKVIDTNCINVIQPDGSYRSEELNLVIIGPSADIIQFECKSFGHEVLNLTRNGYGSTQYIRFSPDFTFGFEESLEVDTNPLLGAGKFATDPAVTLAHQLIYAGHRLYGIAINPNRVFKVNTNAYYEMSGLEVSFEELRTFGGHDAKFIDSLQENEFRLYYYNKFKDIASTLNKAKSIVGTTASLQYMKNVFKEKYLLSEDTSGKFSVDKLKFDKLYKMLTEIYTEDNFVKFFKVLNRKTYLNFDKAVFKINIVPKVNYTIYDGFNLRNTNLAANFNGQNTEINNMNFTKLKNFTGLFEFYKLLCVRGIITSKTKSLDKGYNKALNDLCIKVNNWDLFFSPSEDNFTNDLNKGEEITSDTNIEAAEENISLDLIQQYYLTFNFDNEPENISIENLSSDIIGQLELMPNIERFPNGKKYELDKYTMFHYLRAQEFEHGKSRIALTNSVNEALLNPSRVYTFFSSDYVKKVNKATEAAMFLGWVEQLVYDFTDETSEVSTTDKIADITIIIPYIGPALNIGNMLYKDDFVGALIFSGAVILLEFIPEIAIPVLGTFALVSYIANKVLTVQTIDNALSKRNEKWDEVYKYIVTNWLAKVNTQIDLIRKKMKEALENQAEATKAIINYQYNQYTEEEKNNINFNIDDLSSKLNESINKAMININKFLNQCSVSYLMNSMIPYGVKRLEDFDASLKDALLKYIYDNRGTLIGQVDRLKDKVNNTLSTDIPFQLSKYVDNQRLLSTFTEYIKNIINTSILNLRYESNHLIDLSRYASKINIGSKVNFDPIDKNQIQLFNLESSKIEVILKNAIVYNSMYENFSTSFWIRIPKYFNSISLNNEYTIINCMENNSGWKVSLNYGEIIWTLQDTQEIKQRVVFKYSQMINISDYINRWIFVTITNNRLNNSKIYINGRLIDQKPISNLGNIHASNNIMFKLDGCRDTHRYIWIKYFNLFDKELNEKEIKDLYDNQSNSGILKDFWGDYLQYDKPYYMLNLYDPNKYVDVNNVGIRGYMYLKGPRGSVMTTNIYLNSSLYRGTKFIIKKYASGNKDNIVRNNDRVYINVVVKNKEYRLATNASQAGVEKILSALEIPDVGNLSQVVVMKSKNDQGITNKCKMNLQDNNGNDIGFIGFHQFNNIAKLVASNWYNRQIERSSRTLGCSWEFIPVDDGWGERPL
Supplementary Figure 1. Amino acid sequence of the BoNT/A(0) used in this study.
Amino acid sequence of the BoNT/A(0) used in this study, with the mutated residues that render it catalytically inactive highlighted in red.
